# Supplementary material for: AI-2 production in Fusobacterium nucleatum is subspecies-specific and uncoupled from quorum sensing
Source: J Bacteriol. 2026 Jul 20;208(8):e00223-26. doi: 10.1128/jb.00223-26 (PMC13492134; doi:10.1128/jb.00223-26)
Supplement: Supplementary file 1 [file jb.00223-26-s0001.docx]

**AI-2 Production in *Fusobacterium nucleatum* Is Subspecies-Specific and Uncoupled from Quorum Sensing**

**Bibek G C, Shiqi Xu, Truman Tran & Chenggang Wu^†^**

*Department of Microbiology & Molecular Genetics, the University of Texas Health Science Center, Houston, TX, USA*

**^†^** To whom correspondence should be addressed. Tel. (+1) 713 500 5437; E-mail: [chenggang.wu@uth.tmc.edu](mailto:chenggang.wu@uth.tmc.edu)

Running Title: Subspecies-Specific AI-2 Production in *F. nucleatum*

Keywords: *Fusobacterium nucleatum*, autoinducer 2, *luxS*, activated methyl cycle

**Supporting Tables**

**Table S1:** Bacterial strains and plasmids used in this study.

| **Strain & Plasmid** | **Description** | | **Reference** |
| --- | --- | --- | --- |
| **Strains** |  | |  |
| *Fn* subsp. *nucleatum* 23726 | FNN strain (wild-type strain) | | From ATCC |
| *FNN* CW1 | ∆*galk;* an isogenic derivative of 23726 | | (1) |
| *Fn* subsp. *nucleatum* 25586 | FNN strain (wild-type strain) | | From ATCC |
| *Fn* subsp. *nucleatum* CTI-2 | FNN strain | | (2) |
| *Fn* subsp. *vincentii* 51190 | FNV strain | | From ATCC |
| *Fn* subsp. *vincentii* 49256 | FNV strain | | From ATCC |
| *Fn* subsp. *polymorphum 10953* | FNP strain | | From ATCC |
| *Fn* subsp. *polymorphum 12230* | FNP strain | | (3) |
| *Fn* subsp. *animalis* 51191 | FNA strain | | From ATCC |
| *Fn* subsp. *animalis* 7_1 | FNA strain | | (4) |
| *F. periodonticum* 33693 | Fp strain | | From ATCC |
| *Fn* subsp. *nucleatum* BG26-1 | *WT*::*luc*; an isogenic derivative of 23726 | | This study |
| *Fn* subsp. *nucleatum* BG26-2 | ATCC 25586 with plasmid pLuxS_7_1_ | | This study |
| *Fn* subsp. *animalis BG*26-3 | Deletion of *luxS* in FNA strain 7_1 | | This study |
| *Fn* subsp. *animalis BG*26-3C | *BG*26-3 with pLuxS_7_1_ | | This study |
| *Fn* subsp. *nucleatum* BG26-4 | ∆*metK* pSX11E-P*fdx-E-metK* | | This study |
| *V. harveyi BB170* | AI-2 reporter | | (5) |
| *V. harveyi BB152* | Produce high AI-2 as a positive control | | (5) |
| *E. coli* SZU604 | Expression of 3-methyltransferase from ATCC25586 | | (6) |
| *E. coli* BL21 | Wild-type strain | | NEB |
| *E. coli BL21 (∆luxS)* | *luxS* deletion in *E.coli* BL21 | | (7) |
| *E. coli* DH5a | Cloning host | | NEB |
| **Plasmids** |  | |  |
| pBCG11 | *E. coli*/*Fusobacterium* shuttle vector, chloramphenicol /thiamphenicol resistance; cm^R^/thia^R^ | | (8) |
| pBCG02 | Deletion plasmid with HicA as the selection marker | | (9) |
| pBCG02-*luxS_7_1_* | In-frame insertion of *luxS* from FNA 7_1 into ATCC 23726 | | This study |
| pLuxS_7_1_ | pBCG11 with luxS7_1 expression under the control of *uraA* promoter | | This study |
| pVoPO-04 | A deletion plasmid with *tetR-mazF* | | (10) |
| pBCG10 | A conditional plasmid for F. nucleatum with *mazF* as a counterselection marker | | This study |
| pBCG10-*∆luxS* | *luxS* deletion plasmid | | This study |
| pCM-galK | Suicide plasmid with GalK as a counterselection marker | | (11) |
| pCM-galK-∆*metK* | Metk deletion plasmid for ATCC 23726 | | This study |
| pSX11E | catP gene was replaced in pBCG11 with ermF/AM from pG106 | | This study |
| pSX11E-P*fdx-E-metK* | metK expression is under control by promoter fdx and riboswitch E | | This study |
|  |  |  | |
|  |  |  | |
|  |  |  | |
|  |  |  | |

**Table S2:** Primers and DNA oligos used in this study

| **Primer** | **Sequence^1^** | **Use for** |
| --- | --- | --- |
| BCG02Mch-F | AAACGACATTGCAACACCATTGCTAAGTG | pBCG02-*luxS_7_1_* |
| BCG02Mch-R | GGACGAGCTGTACAAGTAGTAGGG | pBCG02-*luxS_7_1_* |
| uraA-upF | GCTGGAGTGTGATATGTATGGAAGCTACGGTACCGGACTAACAACAGTTGGAACAG | pBCG02-*luxS_7_1_* |
| uraA-upR | CATAAATCCTCTTTTCTTATTTATCTTCTGGTAAAATTTTATTTAAAACTATTCC | pBCG02-*luxS_7_1_* |
| luxS_7_1_-F | AATTTTACCAGAAGATAAATAAGAAAAGAGGATTTATGGAGAAAATAGC | pBCG02-*luxS_7_1_* |
| luxS_7_1_-R | CATATTACCTCTTTCTTAGTAGAATGGTAAAAATAATTAAAAAATAAA | pBCG02-*luxS_7_1_* |
| pepF-dnF | ATTTTTACCATTCTACTAAGAAAGAGGTAATATGAAAGATAGAAAATCA | pBCG02-*luxS_7_1_* |
| pepF-dnR | TCTAGGACGTCGACTTTTGGATCCTTTTAAGCTTGCTGAACTTGCAAATGAAGTTG | pGBC02-*luxS_7_1_* |
| pBCG11-F | TTAAAAAGACTTTAGAAGAATTAAAAGCG | p*luxS*_7_1_ |
| pBCG11-R | TAGCTGATGGTGCTAAAAAAATAAACTAGG | p*luxS*_7_1_ |
| pfdx-F | CCTAGTTTATTTTTTTAGCACCATCAGCTAGTGTAGTAGCCTGTGAAATAAGT | p*luxS*_7_1_ |
| pfdx-R | CCGGAACCTATAGTGAGTCG | p*luxS*_7_1_p*luxS*_7_1_ |
| luxS_7_1_-F2 | AATTTTACCAGAAGATAAATAAGAAAAGAGGATTTATGGAGAAAATAGC | p*luxS*_7_1_ |
| luxS_7_1_-R2 | CTTTCATATTACCTCTTTCTTAACTAAACCTCCTCACTACCTTCTTTTAC | p*luxS*_7_1_ |
| pZP06-nosacB-F | ACCAATAAAAAACGCCCGGCGGTAAACATAGCTGATGGTGCTAAAAAAATAAACTAGG | pBCG10 |
| pZP06-nosacB-R | TAAAATAGAGGCTATAAATAGCGTTAAAAAGACTTTAGAAGAATTAAAAG | pBCG10 |
| tetR-mazF-F | TAGCACCATCAGCTATGTTTACCGCCGGGCGTTTTTTATTGGTGAGAAT | pBCG10 |
| tetR-mazF-R | ATTCTTCTAAAGTCTTTTTAACGCTATTTATAGCCTCTATTTTATTTTATATTT | pBCG10 |
| pBCG10-F | GCTTTTAATTCTTCTAAAGTCTTTTTAAC | pBCG10-∆*luxs_7_1_* |
| pBCG10-R | GGACGATGGCACATAACAAACGCCGTAAACA | pBCG10-∆*luxs_7_1_* |
| luxSupF | CGTTAAAAAGACTTTAGAAGAATTAAAAGCGGAACTTTGGTTGGAA | pBCG10-∆*luxs_7_1_* |
| luxSupR | TGCCACATTCCACAGGATTAACTTGAAAACTTGCTATTTTCTC | pBCG10-∆*luxs_7_1_* |
| luxSdnF | GTTTTCAAGTTAATCCTGTGGAATGTGGCAATTATTTAG | pBCG10-∆*luxs_7_1_* |
| luxsdnR | TTACTTTTTTCAATTTTTTCTAATTCAATCGTGCCAAAGTAAATACATCATCC | pBCG10-∆*luxs_7_1_* |
| 2pCM-galK-F | CTAGGCTAAATATGCTTAAAAGAGatatt | pCM-galK-∆*metK* |
| 2pCM-galK-R | GGGCTTGGTTGTGTTGCTTTTCGGGGCTT | pCM-galK-∆*metK* |
| metKupF(sacI) | ggcg t gagctc AATTTACAGG AATGGAAGGT AAATATGTTC CTG | pCM-galK-∆*metK* |
| metKupR(kpnI) | ggcga GGTACC CTCCAACAAC AACTAATCCA GTAGTACAAA AAACT | pCM-galK-∆*metK* |
| metKdnF(kpnI) | ggcga GGTACC GTATTTGATT TATCTCCAAG AGGAATTGAA AAAGC | pCM-galK-∆*metK* |
| metdnR(SalI) | ggcg a gtcgac TATGATAAAC TTGGATTGCA TTCTAAAAAT TTAT | pCM-galK-∆*metK* |
| ermFAM-F | GTTACAGACAAACCTGAAGTAATAATTTATCTACATTCCCTTTAG | pSX11E |
| ermFAM-R | GTTCATTAGAGCGATAAACTTGAATTTATGGTGCACTCTCAGTACAATCTGCTCTG | pSX11E |
| Re-pBCG11-F | GGAATGTAGATAAATTATTACTTCAGGTTTGTCTGTAACTAAAAACAAGT | pSX11E |
| Re-pBCG11-R | GATTGTACTGAGAGTGCACCATAAATTCAAGTTTATCGCTCTAATGAACAAAGATATT | pSX11E |
| pBCG11-F | TTAAAAAGACTTTAGAAGAATTAAAAGCG | pSX11E |
| pBCG11-R | TAGCTGATGGTGCTAAAAAAATAAACTAGG | pSX11E |
| Pfdx-metK-F | TATACCTAGTTTATTTTTTTAGCACCATCAGCTA GTGTAGTAGCCTGTGAAATAAGTAAG | pSX11E-Pfdx-E-metK |
| Pfdx-metK-R | GTAAAGTATGTAAACTTTTTcatCTTGTTGTTACCTCCTTAGCAGGGTGCTG | pSX11E-Pfdx-E-metK |
| com-metK-F | GGAGGTAACAACAAGatgAAAAAGT TTACATACTT TACATCAG | pSX11E-Pfdx-E-metK |
| com-metK-R | AATCGCTTTTAATTCTTCTAAAGTCTTTTTAA ACCTCTTTTATGTTTGTAACACCAG | pSX11E-Pfdx-E-metK |

**Fig. S1.** **The presence or absence of *luxS* does not affect the growth of the parental strains ATCC 23726 and 7_1.** Indicated strains were inoculated at a 1:20 dilution from overnight cultures into TSPC medium and incubated at 37 °C under anaerobic conditions. Optical density at 600 nm (OD₆₀₀) was measured and recorded hourly.

Fig. S2

**Figure S2. Expression of *luxS* does not alter monospecies biofilm biomass in FNN strains.**
Monospecies biofilms were formed anaerobically in TSPC medium for 72 h in 12-well plates using FNN ATCC 23726 wild type (WT) and the chromosomal *luxS* insertion strain (WT::*luxS*7_1), as well as FNN ATCC 25586 WT and ATCC 25586 carrying a shuttle plasmid expressing *luxS* from FNA strain 7_1 (p*luxS*7_1). Biofilms were stained with 1% crystal violet, washed, and the bound dye was solubilized with 95% ethanol. Biofilm biomass was quantified by measuring absorbance at 580 nm (A580). Data represent mean ± SD from three independent experiments performed in triplicate; no significant differences were detected between WT strains and their corresponding *luxS*-expressing derivatives (n.s.: no significant difference, Student’s *t* test).

**Fig. S3. *Fusobacterium nucleatum* subsp. *polymorphum* strain ATCC 10953 does not produce AI-2.** AI-2 activity in cell-free culture supernatants collected at the stationary phase was measured using the *Vibrio harveyi* BB170 bioluminescence reporter assay. *Escherichia coli* wild type (WT) and its Δ*luxS* mutant served as positive and negative controls, respectively. *F. nucleatum* strains were grown in TSPC medium, and FNP ATCC 10953 was additionally cultured in Schaedler broth supplemented with vitamin K1 (10 µg/mL) to assess the effect of growth medium on AI-2 production. Data are presented as relative luminescence units (RLU; mean ± SD) from three independent experiments, each performed in technical triplicate. The y-axis includes a break to display both low- and high-signal values.

Fig. S4:

**Figure S4. Dose–response calibration of synthetic AI-2 (DPD) in the *Vibrio harveyi* BB170 reporter assay and selection of a working concentration for transcriptomic experiments.**
Cell-free supernatant from *E. coli* BL21 wild type (WT) and its Δ*luxS* mutant were included as positive and negative controls, respectively. Synthetic AI-2 (DPD; dissolved in water) was tested across the indicated concentrations and assayed using the *V. harveyi* BB170 bioluminescence reporter to establish a dose range that yields robust induction comparable to peak activity observed in late-log–phase FNA culture supernatants. Based on this calibration, an intermediate concentration was selected for RNA-seq (used in subsequent treatments of FNN ATCC 23726). Data are shown as relative fluorescence units (RFU; mean ± SD) from technical triplicates (representative experiment).

**Fig. S5. FNA strains exhibits the weakest coaggregation ability with *Actinomyces oris* MG-**Indicated *Fusobacterium nucleatum* strains were grown overnight in TPSC medium, and *A. oris* MG-1 was grown overnight in HIB broth(12). Cells were harvested by centrifugation and washed twice with coaggregation buffer (CAB; 150 mmol·L⁻¹ NaCl, 1 mmol·L⁻¹ Tris, 0.1 mmol·L⁻¹ CaCl₂, 0.1 mmol·L⁻¹ MgCl₂·H₂O; pH 7.5). Coaggregation assays were performed as previously described with minor modifications(13). Equal volumes of *F. nucleatum* and *A. oris* cell suspensions, adjusted to the same optical density in CAB, were mixed to a final volume of 1200 μL. Optical density was measured immediately after mixing and vortexing for 5 s (OD₀ min). After incubation at room temperature for 5 min, mixtures were centrifuged at low speed (100 × g for 1 min) to pellet coaggregated cells. The optical density of the supernatant was measured (OD₁₀ min), and coaggregation efficiency was calculated as (OD₀ min − OD₁₀ min) / OD₀ min × 100. The data represent three independent replicates.

**Reference:**

1.	Wu C, Al Mamun AAM, Luong TT, Hu B, Gu J, Lee JH, D’Amore M, Das A, Ton-That H. 2018. Forward genetic dissection of biofilm development by *Fusobacterium nucleatum*: novel functions of cell division proteins FtsX and EnvC. MBio 9:10.1128/mbio. 00360-18.

2.	Zhou P, GC B, Wu C. 2025. Development of a conditional plasmid for gene deletion in non-model Fusobacterium nucleatum strains. Applied and Environmental Microbiology:e01816-24.

3.	Han YW, Ikegami A, Rajanna C, Kawsar HI, Zhou Y, Li M, Sojar HT, Genco RJ, Kuramitsu HK, Deng CX. 2005. Identification and characterization of a novel adhesin unique to oral fusobacteria. Journal of bacteriology 187:5330-5340.

4.	Strauss J, Kaplan GG, Beck PL, Rioux K, Panaccione R, DeVinney R, Lynch T, Allen-Vercoe E. 2011. Invasive potential of gut mucosa-derived Fusobacterium nucleatum positively correlates with IBD status of the host. Inflammatory bowel diseases 17:1971-1978.

5.	Taga ME, Xavier KB. 2011. Methods for analysis of bacterial autoinducer-2 production. Curr Protoc Microbiol Chapter 1:Unit1C 1.

6.	Liu L, He Y, Zhang T, Geng R, Hu Y, Luo M, Zhou H, Liu X. 2024. Equip *Fusobacterium nucleatum* genetic tool kits with compatible shuttle vectors and engineered intermediatory E. coli strains for enhanced transformation efficiency. bioRxiv:2024.07. 17.603877.

7.	Han X, Bai H, Tu J, Yang L, Xu D, Wang S, Qi K, Fan G, Zhang Y, Zuo J. 2015. Deletion of luxS further attenuates the virulence of the avian pathogenic *Escherichia coli* aroA mutant. Microbial pathogenesis 88:39-47.

8.	Xu S, GC B, Phan A, Wu C. 2026. The gene encoding ornithine decarboxylase for putrescine biosynthesis is essential for the viability of *Fusobacterium nucleatum*. Journal of Bacteriology 208:e00387-25.

9.	Gc B, Zhou P, Wu C. 2023. HicA Toxin-Based Counterselection Marker for Allelic Exchange Mutations in *Fusobacterium nucleatum*. Applied and Environmental Microbiology:e00091-23.

10.	Ponath F, Zhu Y, Cosi V, Vogel J. 2022. Expanding the genetic toolkit helps dissect a global stress response in the early-branching species *Fusobacterium nucleatum*. Proceedings of the National Academy of Sciences 119:e2201460119.

11.	Nariya H, Miyata S, Suzuki M, Tamai E, Okabe A. 2011. Development and application of a method for counterselectable in-frame deletion in Clostridium perfringens. Applied and environmental microbiology 77:1375-1382.

12.	Wu C, Huang IH, Chang C, Reardon‐Robinson ME, Das A, Ton‐That H. 2014. Lethality of sortase depletion in *A ctinomyces oris* caused by excessive membrane accumulation of a surface glycoprotein. Molecular microbiology 94:1227-1241.

13.	Kaplan CW, Lux R, Haake SK, Shi W. 2009. The *Fusobacterium nucleatum* outer membrane protein RadD is an arginine‐inhibitable adhesin required for inter‐species adherence and the structured architecture of multispecies biofilm. Molecular microbiology 71:35-47.
